# Serum miR-1228-3p and miR-181a-5p as Noninvasive Biomarkers for Non-Small Cell Lung Cancer Diagnosis and Prognosis

**DOI:** 10.1155/2020/9601876

**Published:** 2020-07-06

**Authors:** Wei-Xiao Xue, Meng-Yu Zhang, Rui Li, Xiao Liu, Yun-Hong Yin, Yi-Qing Qu

**Affiliations:** ^1^Department of Pulmonary and Critical Care Medicine, Qilu Hospital, Cheeloo College of Medicine, Shandong University, Jinan 250012, China; ^2^Department of Critical Care Medicine, Chui Yang Liu Hospital affiliated to Tsinghua University, Beijing 100022, China; ^3^Department of Pulmonary and Critical Care Medicine, Qilu Hospital of Shandong University, Jinan 250012, China

## Abstract

**Background:**

Lung cancer is the leading cause of cancer-related mortality worldwide, and non-small cell lung cancer (NSCLC) accounts for over 80% of all lung cancers. Serum microRNAs (miRNAs), due to their high stability, have the potential to become valuable noninvasive biomarkers. This present study was aimed to identify the serum miRNAs expression signatures for the diagnosis and prognosis of NSCLC using bioinformatics analysis.

**Methods:**

A total of 12 miRNAs profiling studies have been identified in Pubmed, Gene Expression Omnibus (GEO), and ArreyExpress databases. Differentially expressed miRNAs (DEmiRNAs) were analyzed according to GEO2R online tool and RRA method from R. Then, prediction of DEmiRNAs' target genes from TargetScan, PicTar, miRDB, Tarbase, and miRanda database. Furthermore, we using reverse transcription– quantitative polymerase chain reaction (RT-qPCR) to evaluate the expression levels of DEmiRNAs in serum samples obtained from NSCLC patients and healthy controls. Subsequently, the clinical significance of the tested miRNAs was determined using receiver operating characteristic (ROC) analysis and Cox regression analysis.

**Results:**

A total of 27 DEmiRNAs were identified and 5 of them (miR-1228-3p, miR-1228-5p, miR-133a-3p, miR-1273f, miR-545-3p) were significantly up-regulated and 4 of them (miR-181a-5p, miR-266-5p, miR-361-5p, miR-130a-3p) were significantly down-regulated in NSCLC patients compared with healthy controls. RT-qPCR validated that miR-1228-3p (*P* =0.006) and miR-181a-5p (*P* =0.030) were significantly differentially expressed in the serum of NSCLC patients and healthy controls. ROC analysis on miR-1228-3p and miR-181a-5p revealed the area under the curve (AUC) of 0.685 (95% confidence interval [CI], 0.563–0.806; *P* =0.006) and 0.647 (95% CI, 0.506–0.758; *P* =0.049). ROC analysis on miR-1228-3p combined miR-181a-5p revealed the AUC of 0.711 (95% CI, 0.593–0.828; *P* =0.002). Multivariate Cox regression analysis demonstrated that the high serum miR-1228-3p level was an independent factor for the poor prognosis of NSCLC patients.

**Conclusions:**

Serum miR-1228-3p and miR-181a-5p are potential noninvasive biomarkers for the diagnosis and prognosis of NSCLC patients.

## 1. Background

Lung cancer remains the leading cause of cancer-associated mortality worldwide, of which NSCLC accounts for over 80% of lung cancer-related deaths [1, 2]. Despite improvements in the chemotherapeutic drugs used over time, the 5-year survival rate of NSCLC patients is only 18% [3]. Besides, surgical resection is the most effective treatment for NSCLC, but most newly diagnosed patients are at the onset of advanced or metastatic stages and usually lost the chance for operation. Low-dose computed tomography (LDCT) provides excellent anatomic information in the diagnosis of early NSCLC patients. However, LDCT still have a few limitations including high false-positive rates, potential over-diagnosis, excessive cost and the potential harm related to radiation exposure. Furthermore, the response rates in subsets of NSCLC with tyrosine kinase receptors (mutant EGFR, ALK, and ROS1) were high, drug resistance has been a major challenge [4–6]. Therefore, it is vital to find an early and accurate way to diagnosis and enhance patient's chances to receive proper treatments.

Currently, considerable studies revealed miRNAs as a new opportunity in the field of noninvasive diagnosis. MiRNAs are endogenous 20–25 nucleotides long, have been found to have a profound impact on several biological and pathological processes like cell development, differentiation, proliferation and apoptosis, which play important roles in the carcinogenesis and progression of lung cancer [7, 8]. DEmiRNAs in NSCLC tissue and adjacent nontumor tissues have been reported in a previous study [9]. Circulating miRNAs also could be potential and promising biomarkers for the diagnosis and prognosis of NSCLC. However, the data from different studies are quite variable. Therefore, identification of specific circulating miRNAs reflecting investigated pathological conditions may help to develop novel noninvasive biomarkers and shed a new light on molecular processes involved in cancer and a systematical analysis of miRNA expression signature from multiple platforms and multicenter NSCLC studies is urgently needed.

In this study, due to the presence and stability of cell-free miRNAs have been clearly demonstrated in all body fluid [10, 11], we identified serum and plasma miRNAs related to NSCLC, and then screened and validated miR-1228-3p and miR-181a-5p expression level in the serum of NSCLC patients in comparison to serum of healthy volunteers.

## 2. Methods

### 2.1. Data Collection

Up to January 1, 2018, a total of 3 databases including Pubmed (http://www.ncbi.nlm.nih.gov/), GEO (http://www.ncbi.nlm.nih.gov/geo/) and ArrayExpress (http://www.ebi.ac.uk/arrayexpress/) were used for literature retrieval, and the search terms were (miR-∗ OR miRNA OR microRNA) AND (lung AND (tumor OR cancer OR carcinoma)). The selection criteria for the literature were: miRNAs detection was microarray or miRNAs sequencing; studies were published in English; patients had pathologically confirmed NSCLC; patients had no history of other cancers; none of the patients received preoperative treatment, such as radiotherapy or chemotherapy; control group was healthy normal controls; the experimental samples were derived from serum or plasma.

### 2.2. Identification of DEmiRNAs

GEO2R (https://www.ncbi.nlm.nih.gov/geo/geo2r/) is a web tool for screening DEmiRNAs by comparing two groups of samples. The procedure of GEO2R is the following: firstly, enter a series accession number in the box. Then, click “Define groups” and enter names (NSCLC and healthy control) for the groups of samples you plan to compare. After samples have been assigned to groups, click “Top 250” to run the test with default parameters. To see more than the top 250 results, or if you want to save the results, the complete results table may be downloaded using the “Save all results” button. The cut-off criterion was set as the *P* <0.05 and absolute fold change (FC) >1.5. In addition, the R package ggplot2 package (version 2.2.1, https://cran.r-project.org/web/packages/ggplot2) was used to perform the volcano plots of all the miRNAs among 12 miRNAs profiling. Moreover, heat maps for the DEmiRNAs was generated using the pheatmap package (version 1.0.8, https://cran.r-project.org/web/packages/pheatmap). For some literatures that did not find original data, we used the miRNAs data listed in the paper or miRNAs information in supplementary data for analysis. All miRNAs names are standardized through miRBase.

### 2.3. Target Gene Prediction and Functional Enrichment Analysis

Target genes of DEmiRNAs were predicted by 4 different online databases including TargetScan (http://www.targetscan.org/), PicTar (http://pictar.mdc-berlin.de/), miRanda (http://www.miranda-im.org/) and miRDB (http://mirdb.org/). The target genes were screened by the intersection of TargetScan, PicTar, miRanda and miRDB. Then TarBase (http://www.microrna.gr/tarbase/) was used to validate the target genes. Then all of the target genes were sorted from the union of the front genes and the validation genes. Venn Diagram package (version 1.6.17, https://cran.r-project.org/web/packages/VennDiagram/) were applied to identify the overlapping target genes of DEmiRNAs among 12 miRNAs profiling. Furthermore, GeneCodis web tool (http://genecodis.cnb.csic.es) was used to function enrichment analysis [12–14]. The resulting gene list was submitted to GeneCodis in order to identify the targeted pathway, threshold of FDR was 0.05 and considering enrichment in Panther and Kyoto Encyclopedia of Genes and Genomes (KEGG) pathways.

### 2.4. Patients and Samples

For the current study, we recruited 50 patients with first diagnosis of NSCLC and previously untreated from the Qilu Hospital of Shandong University, from July 2017 to December, 2017. Moreover, control group consist of thirty healthy volunteers (well matched to the patients according to age and gender) was screened from the Qilu Hospital of Shandong University. This study was approved by the Ethics Committee of Qilu Hospital of Shandong University (KYLL-2013-097; 25 February 2014), and written informed consent was obtained from all patients or their guardians. Once the patient was diagnose with NSCLC, about 5 ml of peripheral blood was collected in a sterile tube without anticoagulant before any treatment was performed, and allowed to stand at room temperature for 30 – 60 min to clot, then samples were centrifuged at 4000 rpm for 15 min at the room temperature and for another 10 min at 12000 rpm at 4°C to completely remove the cell debris. Finally, the resultant serum was stored at -80°C, samples with visual evidence of hemolysis were excluded from further analysis.

### 2.5. Total miRNA Isolation and miRNAs Expression Analysis by RT-qPCR

Total miRNA was extracted from 200 *μ*L of serum samples using miRcute serum/plasma miRNA isolation kit (Tiangen Biotech) according to the manufacturer's protocol. The concentration of miRNA was measured using the NanoDLite. According to the manufacturer's instructions, miRNA profiling was performed with RT-qPCR instrument StepOnePlus™ Real—Time PCR System (Thermo Fisher Scientific) using miDETECT A TrackTM miRNART- qPCR Starter Kit (RiboBio). The primers of these miRNAs and cel-miR-39 were obtained from RiboBio Corporation (Guangzhou, China). After the reactions, the *Δ*Ct values were determined. The fold change of each miRNA expression was calculated using the 2^-*ΔΔ*CT^ method [15].

### 2.6. Statistical Analysis

The serum miRNA level was expressed as 2^-*ΔΔ*CT^ to maintain the normal distribution of the parameter and assure a positive correlation with the miRNA level of expression and student's t test was used to analyze miRNA expression level. Mann–Whitney tests were used to check associations between miRNA expression levels and clinicopathological features of the patients. The survival rates were estimated by the Kaplan-Meier analysis and the significance of differences was examined by log-rank test. We also performed overall survival (OS) to investigate survival outcome. OS was defined as the time between the date of surgery and the date of death or last followup. The diagnostic performance of miRNAs was assessed by the ROC curve analysis and calculated the AUC to evaluate the predictive power of candidate miRNAs for NSCLC. Multivariate analysis of the prognostic factors was performed with Cox regression model. Data was presented as mean ± standard deviation (SD) and *P* <0.05 were considered statistically significant. All statistical analysis was performed using the SPSS version 20.0 (IBM Corporation, Armonk, NY, USA) and GraphPad Prism 6.0 (GraphPad Software, Inc., La Jolla, CA, USA).

## 3. Results

### 3.1. Identification of DEmiRNAs

**Figure 1:**
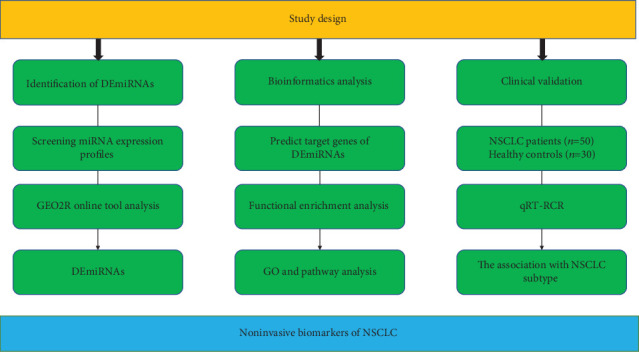
The general overview of study design.

**Figure 2:**
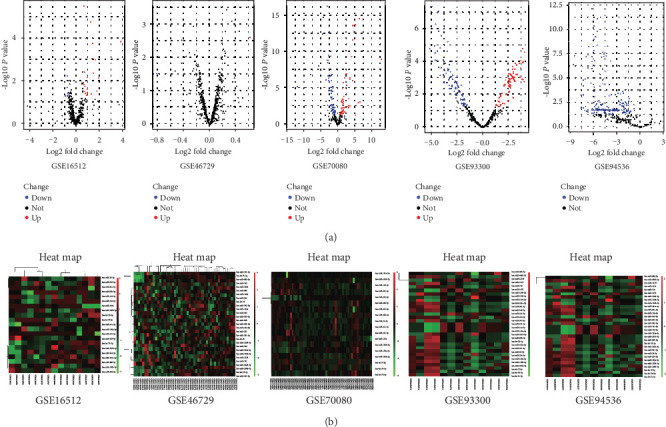
Data analysis of 5 GEO chips. (a) Volcano plots of the different miRNA expression analysis. *x*-axis: log 2 fold change; *y*-axis: -log10 *p*-value for each probes. (b) Two-dimensional hierarchical clustering of 27 DEmiRNA s in all samples. MiRNAs are in rows; samples are in columns.

The general overview of the study design is shown in Figure 1. According to the selection criteria, 12 full-text studies were retrieved from July 2009 to January 2018 (Table 1) [11–18]. All these 12 studies including 413 NSCLC patients and 513 healthy controls were used to screen miRNA signature and a total of 2381 significantly up-expressed and 513 significantly down-expressed miRNAs were extracted. Then 5 GEO datasets (GSE16512, GSE70080, GSE93300, GSE94536 and GSE46729) were used to perform volcano maps and to get the DEmiRNAs (*P* <0.05 and absolute FC >1.5, Figure 2). According to RRA method 5 up-regulated miRNAs and 27 down-regulated miRNAs were screened (FDR<0.05). Finally we selected 9 DEmiRNAs including the top 4 down-regulated miRNAs (hsa-miR-181a-5p, hsa-miR-26b-5p, hsa-miR-361-5p and hsa-miR-130a-3p) and all 5 up-regulated miRNAs (hsa-miR-1228-3p hsa-miR-1228-5p hsa-miR-133a-3p hsa-miR-1273f hsa-miR-545-3p) for the further study based on the FDR value.

### 3.2. Target Gene Prediction and Functional Enrichment Analysis

**Figure 3:**
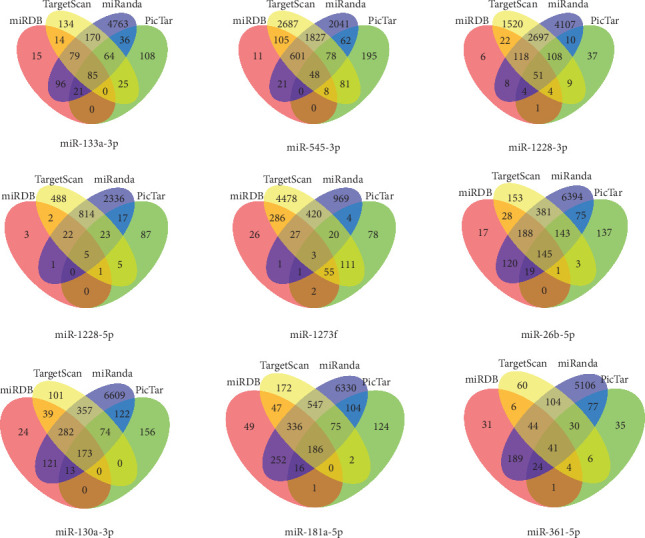
Overlap of 9 DEmiRNAs target gene prediction using 4 miRNA prediction databases.

**Figure 4:**
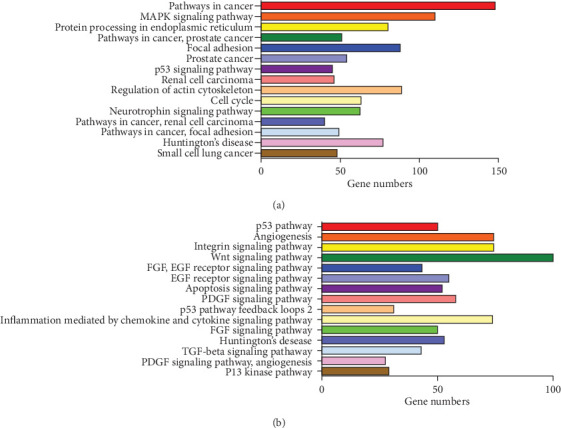
Target gene networks identified through KEGG (a) and pather (b) pathway analysis.

TargetScan, miRanda, miRDB and PicTar were used to predict target genes of 9 DEmiRNAs, overlap predicated target genes from 4 databases (Figure 3) and Tarbase was used to validate target genes. Then, the overlapped genes plus validated genes was defined as target genes. Furthermore, removing 108 repeated target genes, we gained a total of 8002 target genes of DEmiRNAs (Table 2). 8002 target genes were used to perform functional enrichment analysis. The gene ontology (GO) analysis showed that these target genes were mainly involved in the regulation of transcription, DNA-dependent (GO:0006355), Nucleus (GO:0005634) and Protein binding (GO:0005515) (Table 3). Further Panther and KEGG pathway analysis were performed to investigate the significance of target genes in the development of NSCLC, the results showed that these genes were significantly enriched in Pathways in cancer and Wnt signaling pathway (Figure 4).

### 3.3. Association between Expression Levels of Serum miRNAs and Clinicopathological Characteristics

Among the 50 NSCLC patients, there were 34 males and 16 females, 37 adenocarcinomas (ADCs) and 13 squamous cell carcinomas (SCCs), 14 patients are at stage I, 7 at stage II, 10 at stage III and 19 at stage IV. The clinicopathological characteristics of patients with NSCLC and healthy volunteers are presented in Table 4.

**Figure 5:**
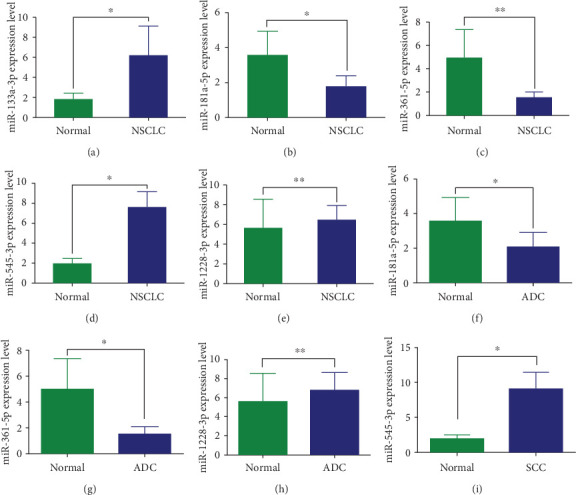
(a-e) The relative expression of 5 DEmiRNAs in NSCLC group and normal control group. (f-h) The expression levels of 3 DEmiRNAs in ADC group and normal control group. (i) The expression level of 1 DEmiRNA in SCC group and normal control group. ∗*P*<0.05, ∗∗*P*<0.01.

The current study revealed that miR-1228-3p, miR-133a-3p and miR-545-3p were significantly up-regulated (*P* =0.006, *P* =0.043 and *P* =0.047, respectively), while miR-181a-5p and miR-361-5p were significantly down-regulated (*P* =0.029 and *P* =0.006) in NSCLC patients compared with healthy controls (Figure 5(a)-5(e)). Among NSCLC patients, miR-1228-3p expression level (*P* =0.009) in ADC patients was higher compared with healthy controls, while the expression levels of miR-181a-5p (*P* =0.031) and miR-361-5p (*P* =0.006) were lower than healthy controls (Figure 5(f)-5(h)). In SCC patients, miR-545-3p expression level (*P* =0.034) was higher compared with healthy controls (Figure 5(i)).

**Figure 6:**
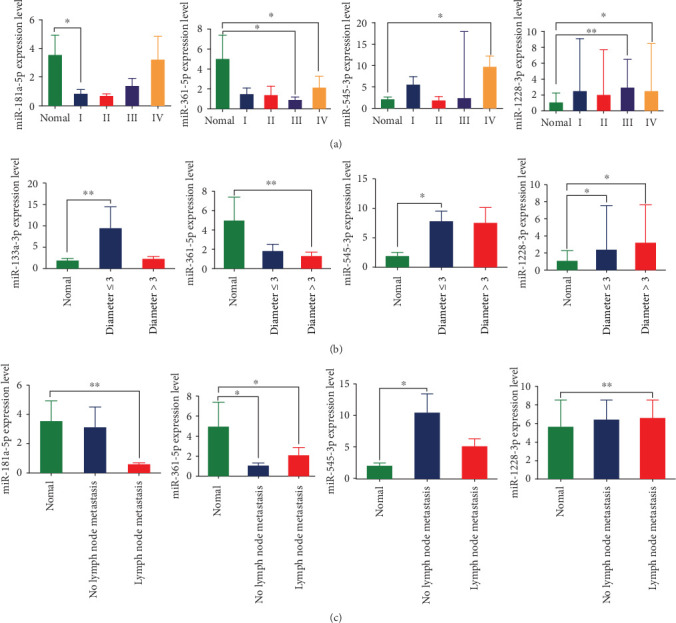
(a) The relative expression level of miRNAs between different TNM stages of NSCLC group and normal control group; (b) The relative expression level of miRNAs between normal control group and NSCLC with different tumor size; (c) The relative expression level of miRNA between normal control group and NSCLC group with or without lymph node metastasis. ∗*P*<0.05, ∗∗*P*<0.01.

As for TNM stage, the expression level of miR-181a-5p and were significantly lower than healthy controls in TNM stage I (*P* =0.028), as well as miR-361-5p in both TNM stage III (*P* =0.016) and IV (*P* =0.048). On the contaray, the expression level of miR-1228-3p in TNM stage III (*P* =0.007) and IV (*P* =0.026) were significantly higher compared with healthy controls, as well as miR-545-3p in TNM stage IV (*P* =0.013) (Figure 6(a)). When the tumor diameter < =3 cm, the expression levels of miR-1228-3p, miR-133a-3p and miR-545-3p were significantly higher compared with healthy controls (*P* =0.020, *P* =0.005 and *P* =0.037) and miR-1228-3p expression level in tumor diameter>3 cm group was significantly higher compared with healthy controls (*P* = 0.017). In contrast, the expression level of miR-361-5p in tumor diameter>3 cm group was significantly lower compared with healthy controls (*P* =0.006) (Figure 6(b)). The high expression level of miR-1228-3p and low expression level of miR-181a-5p were related to lymph node metastasis (*P* =0.005, *P* =0.003, respectively), while the high expression level of miR-545-3p was related to no lymph node metastasis (*P* =0.045). As for miR-361-5p, its expression level in no lymph node metastasis group was lower compared with healthy controls (*P* =0.027), as well as lymph node metastasis group compared with healthy controls (*P* =0.015) (Figure 6(C)).

### 3.4. Diagnostic Value of Serum miRNAs NSCLC Patients

ROC curve analysis was used to investigate the diagnostic value of miR-1228-3p, miR-133a-3p, miR-545-3p, miR-181a-5p and miR-361-5p in distinguishing NSCLC patients from normal controls. Expression levels of 5 serum miRNAs were measured from NSCLC patients and healthy controls. The diagnostic relevance of each miRNA, both single and combination, were analyzed (Table 5). The results showed that ROC analysis revealed the AUC for miR-1228-3p was 0.685 (95% confidence interval [CI], 0.563-0.806; *P* =0.006), for miR-133a -3p was 0.636 (95% CI, 0.512-0.760; *P* =0.043), for miR-545-3p was 0.635 (95% CI, 0.514-0.756; *P* =0.045), for miR-181a-5p was 0.647 (95% CI, 0.506-0.758; *P* =0.049) and the AUC for miR-361-5p was 0.635 (95% CI, 0.508-0.761; *P* =0.045). ROC analysis indicated that the combination of miR-1228-3p and miR-181a-5p provided best diagnostic discriminant with an AUC of 0.711(95% CI 0.593-0.828; *P* =0.002).

### 3.5. Associations of Serum miRNAs Expression Levels with OS

**Figure 7:**
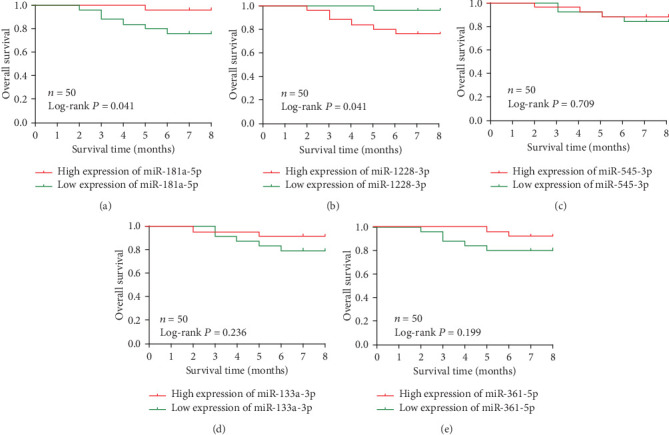
Kaplan-Meier survival curves by different miRNA expression levels of of miR-181a-5p, miR-1228-3p, miR-545-3p, miR-133a -3p and miR-361-5p in an independent NSCLC chort. (a) OS between low and high miR-181a-5p expression. (b) OS between low and high miR-1228-3p expression. (c) OS between low and high miR-545-3p expression. (d) OS between low and high miR-133a -3p Aexpression. (e) OS between low and high miR-361-5p expression.

To explore whether serum miRNAs expression levels will affect the clinical outcomes, we constructed a prognostic classifier using Kaplan-Meier analysis on 50 NSCLC patients. It showed that miR-1228-3p and miR-181a-5p expression levels were significantly associated with the OS of NSCLC patients (both *P* =0.041) (Figure 7). As for miR-133a -3p, miR-545-3p and miR-361-5p, the expression levels of all these 3 miRNAs have no significance with OS statistically (*P* =0.236, *P* =0.709, *P* =0.199, respectively). The median OS in miR-133a -3p, miR-545-3p and miR-361-5p low expression group were both 8 months whereas in high expression group were all 7 months. The multivariate Cox hazard regression analysis demonstrated that expression level of serum miR-1228-3p were an independent prognostic indicator of NSCLC (hazard ratio(HR) 1.487, 95% CI 1.130-1.958; *P* =0.005).

## 4. Discussion

In the current study, we integrated expression profiles of 413 NSCLC patients and 513 healthy controls in 5 datasets from GEO database and identified a panel of 32 DEmiRNAs. According to FDR value, we finally identified 9 DEmiRNAs for further study. Then we used 5 online databases and screened a total of 8002 target genes of these 9 DEmiRNAs, functional enrichment analysis showed that these target genes were mainly involved in the regulation of transcription, DNA-dependent, Nucleus, Protein binding and significantly enriched in Pathways in cancer, especially in Wnt signaling pathway. The high expression levels of miR-1228-3p, miR-133a-3p and miR-545-3p and low expression levels of miR-181a-5p and miR-361-5p were also validated via an independent NSCLC cohort from Qilu Hospital of Shandong University. The result indicated that the expression level of miR-1228-3p was related to TNM stage, tumor diameter and lymph node metastasis, the expression level of miR-545-3p was related with TNM stage and tumor diameter, the expression levels of miR-181a-5p and miR-361-5p were related to TNM stage and lymph node metastasis, the expression level of miR-133a-3p was related with tumor diameter only. Furthermore, the expression levels of miR-1228-3p and miR-181a-5p were significantly associated with the OS of NSCLC patients.

The incidence of lung cancer is the leading factor in malignant tumors. Up to date, the gold standard in diagnosing NSCLC is pathologic evidence of malignant cells, which typically requires a surgical procedure or an invasive examination. It is mostly at advanced stage as long as lung cancer is diagnosed. The 5-year survival rate of advanced lung cancer is less than 20%, but the 5-year survival rate of stage IA lung cancer can reach 60% [21]. Early diagnosis is the key strategy to improve the outcome of lung cancer. Current methods including CEA level and CT screening cannot predict the risk of NSCLC for patients who have small lung nodules accurately. Therefore, specific and sensitive biomarkers for the detection of malignancies are urgently required to reduce the worldwide morbidity and mortality caused by NSCLC.

MiRNAs have been identified as potential biomarkers for lung cancer, it can be used to evaluate the invasion, metastasis, treatment response and prognosis of cancer. Although tumor sample miRNAs have been demonstrated to be associated with the development of tumors in many studies, it is difficult to obtain tumor samples in clinical practice. Recent studies have supported that circulating miRNAs have potential diagnostic effects for NSCLC. Studies [22, 23] have shown that there is a significant difference between serum miRNAs and blood cell miRNAs in patients with lung cancer, and blood cells can affects the detection rate of whole blood miRNAs [18, 24]. So that we choose serum miRNAs as the source of hematology of the subjects. Numerous circulating miRNA signatures have been reported for the detection of NSCLC, but the miRNAs signature identified by different groups vary from one another because of the inconsistencies platforms, it is necessary to find a better way to screen different miRNAs. The RRA approach is as good way to eliminate differences among various platforms, by which reordered miRNAs according to the FDR value.

MiR-1228 is located in the LRP1 gene on chromosome 12. This gene is mainly involved in basic metabolism and cell structure, which is a key component of maintaining cell survival [25]. There are many researches of miR-1228-3p in various diseases. It has been reported that miR-1228-3p expression level was involved in drug resistant of breast cancer, chronic heart failure, endometrial carcinoma, it can be expressed steadily in the prostate cancer, colorectal cancer and secretions of hepatocellular carcinoma [26–31]. There are another two studies about miR-1228-3p on NSCLC. One is about miR-1228-3p differentially expressed in NSCLC exocrine [32] and another suggested that miR-1228-3p can be used as an endogenous reference gene [24]. It means that miR-1228-3p can be stable in the exocrine and circulatory and further confirms that it can be released to the cell through the exocrine.

The miR-181 family includes miR-181a, miR-181b, miR-181c and miR-181d, contains the same seed sequence, which can display the functional redundancy of the gene in mRNA [33]. The role of miR-181a-5p as a tumor suppressor has been confirmed in previous studies. For example, lower expression level of miR-181a-5p was associated with a worse survival rate in colorectal cancer [34]. In gastric cancer and lung cancer, the expression of miR-181a-5p through target BCL2 increased the sensitivity of cancer cells to cisplatin and vincristine, which further induced the apoptosis of cancer cells [35]. In addition, miR-181a-5p can reduce the metastasis in breast and colon cancer cells [34]. All the results suggested that miR-181a-5p can affect the survival, invasion and metastasis of tumor cells, and even the therapeutic response to chemotherapeutic drugs, while the further role of miR-181a-5p in NSCLC remains to further explore.

## 5. Conclusion

In conclusion, our study indicated that miR-181a-5p play an important role in the early diagnosis of NSCLC and the combined expression levels of miR-1228-3p and miR-181a-5p have certain diagnosis efficancy for NSCLC. Furthermore, high expression level of miR-1228-3p and low expression level of miR-181a-5p have a shorter survival time, which indicated that miR-1228-3p and miR-181a-5p can be used as noninvasive diagnostic and prognostic biomarkers for NSCLC. However, it is vital to conduct more in-depth studies to explore the molecular roles of serum miR-1228-3p and miR-181a-5p in the future.

## Figures and Tables

**Table 1:** The basic information of the 12 studies.

Study	References	Region	MiRNA numbers	Tumor type	Sample resource	Sample numbers	Time	Database
1	Lodes [12]	North America	547	Various	Serum	2 NSCLC 14 normal	2009.07	Pubmed ArrayExpress GSE16512
2	Wang [13]	China	427	Various	Serum	88 NSCLC 17 normal	2011.06	Pubmed
3	Foss [14]	Italy	880	Various	Serum	11 NSCLC 11 normal	2011.03	Pubmed
4	Roth [16]	Germany	1158	Various	Serum	21 NSCLC 11 normal	2012.06	Pubmed
5	Rani [17]	Ireland	667	ADC	Serum	40 NSCLC 40 normal	2013.12	Pubmed
6	Hu [18]	China	723	Various	Plasma	73 NSCLC 34 normal	2014.02	Pubmed
7	Wang [19]	China	754	Various	Serum	31 NSCLC 31 normal	2015.08	Pubmed
8	Nadal [20]	North America	334	Various	Serum	70 NSCLC 22 normal	2015.06	Pubmed
9	Halvorsen AR	Norway	272	Various	Serum	38 NSCLC 16 normal	2016.10	GSE70080
10	Qu LL	China	5915	ADC	Plasma	9 ADC 4 normal	2017.01	GSE93300
11	Liu X	China	5915	Various	Plasma	6 NSCLC 3 normal	2017.02	GSE94536
12	Xu ZL	USA	7815	Various	NSCLC serum Normal plasma	24 NSCLC 24 normal	2017.05	GSE46729

**Table 2:** Target gene prediction of 9 DEmiRNAs.

	4 target gene prediction databases					1 verified database	Target gene numbers
miRNA	TargetScan	Pic tar	mirDB	miRanda	Overlap	Tarbase	Union
miR-1228-3p	4529	224	214	7103	51	3	54
miR-1228-5p	1360	139	34	3218	5	15	20
miR-133a-3p	571	339	310	5314	85	298	383
miR-1273f	5400	274	401	1445	3	0	3
miR-545-3p	5435	472	794	4678	48	499	547
miR-181a-5p	1365	508	887	7846	186	1884	1884
miR-26b-5p	1042	543	508	7465	145	2949	2949
miR-361-5p	295	218	340	5615	41	685	726
miR-130a-3p	1026	578	652	7751	173	1543	1544
Total							8110

**Table 3:** GO analysis of target genes.

Function enrichment	FDR	Target
*Go biological process (BP)*		
GO:0006355: Regulation of transcription, DNA-dependent	4.51E-119	621
GO:0045893: Positive regulation of transcription, DNA-dependent	7.41E-46	204
GO:0045944: Positive regulation of transcription from RNA polymerase II promoter	3.83E-45	232
GO:0006915: Apoptotic process	3.61E-41	229
GO:0045892: Negative regulation of transcription, DNA-dependent	9.24E-39	174
GO:0000122: Negative regulation of transcription from RNA polymerase II promoter	1.65E-37	176
GO:0007165: Signal transduction	2.73E-36	353
GO:0010467: Gene expression	5.03E-34	168
GO:0006468: Protein phosphorylation	2.91E-33	164
GO:0044419: Interspecies interaction between organisms	1.12E-32	144
*Go cellular component(CC)*		
GO:0005634: Nucleus	0	2024
GO:0005737: Cytoplasm	0	1859
GO:0005634: GO:0005737: Nucleus, cytoplasm	5.851E-210	975
GO:0005829: Cytosol	5.38E-155	813
GO:0005730: Nucleolus	5.43E-134	606
GO:0005634: GO:0005730: Nucleus, nucleolus	2.29E-129	584
GO:0005737: GO:0005829: Cytoplasm, cytosol	9.11E-123	606
GO:0005622: Intracellular	1.24E-108	685
GO:0016020: Membrane	9.19E-97	1104
GO:0005654: Nucleoplasm	1.86E-95	394
*Go molecular function(MF)*		
GO:0005515: Protein binding	0	1770
GO:0046872: Metal ion binding	9.92E-150	975
GO:0000166: Nucleotide binding	1.10E-134	771
GO:0003677: DNA binding	7.10E-134	688
GO:0008270: Zinc ion binding	1.24E-115	693
GO:0046872: GO:0008270: Metal ion binding, zinc ion binding	3.22E-108	648
GO:0005524: ATP binding	8.41E-97	550
GO:0005515: GO:0000166: Protein binding, nucleotide binding	6.55E-95	363
GO:0005524: GO:0000166: ATP binding, nucleotide binding	4.73E-89	506
GO:0003677: GO:0005515: DNA binding, protein binding	8.64E-80	278

**Table 4:** The demographic and clinical features of the study.

Characteristic	Healthy control (n =30)	NSCLC (n =50)	*P* value
Age (years)	62 ± 7	62 ± 9	0.292
Gender			0.481
Male	20	34	
Female	10	16	
Smoking status			0.146
Nonsmokers	20	25	
Smokers	10	25	
Type of NSCLC tissue			—
SCC	—	13	
ADC	—	37	
Size of NSCLC			—
Diameter < =3 cm	—	28	
Diameter>3 cm	—	22	
Lymph node metastasis			—
Yes	—	22	
No	—	28	
TNM stage			—
I	—	14	
II	—	7	
III	—	10	
IV	—	19	

**Table 5:** ROC curve analysis of differential miRNAs.

miRNAs	AUC	*P* value	95% CI	
			Lower	Upper
miR-1228-3p	0.685∗	0.006	0.563	0.806
miR-133a-3p	0.636∗	0.043	0.512	0.760
miR-545-3p	0.635∗	0.045	0.514	0.756
miR-181a-5p	0.647∗	0.049	0.506	0.758
miR-361-5p	0.635∗	0.045	0.508	0.761
miR-1228-3p + miR-133a-3p	0.615	0.087	0.490	0.739
miR-1228-3p + miR-545-3p	0.622	0.069	0.500	0.744
miR-1228-3p + miR-181a-5p	0.711∗	0.002	0.593	0.828
miR-1228-3p + miR-361-5p	0.651∗	0.025	0.520	0.781
miR-133a-3p + miR-545-3p	0.705∗	0.002	0.592	0.818
miR-133a-3p + miR-181a-5p	0.679∗	0.008	0.554	0.804
miR-133a-3p + miR-361-5p	0.637∗	0.041	0.510	0.764
miR-545-3p + miR-181a-5p	0.585	0.207	0.460	0.710
miR-545-3p + miR-361-5p	0.611	0.097	0.485	0.738
miR-181a-5p + miR-361-5p	0.646∗	0.030	0.520	0.772
miR-1228-3p + miR-133a-3p + miR-545-3p	0.698∗	0.003	0.585	0.811
miR-1228-3p + miR-133a-3p + miR-181a-5p	0.661∗	0.017	0.535	0.787
miR-1228-3p + miR-133a-3p + miR-361-5p	0.616	0.084	0.489	0.743
miR-1228-3p + miR-545-3p + miR-181a-5p	0.588	0.19	0.463	0.713
miR-1228-3p + miR-545-3p + miR-361-5p	0.609	0.105	0.483	0.735
miR-1228-3p + miR-181a-5p + miR-361-5p	0.647∗	0.029	0.517	0.776
miR-133a-3p + miR-545-3p + miR-181a-5p	0.609	0.103	0.486	0.733
miR-133a-3p + miR-545-3p + miR-361-5p	0.643∗	0.033	0.519	0.768
miR-133a-3p + miR-181a-5p + miR-361-5p	0.663∗	0.015	0.538	0.789
miR-545-3p + miR-181a-5p + miR-361-5p	0.605	0.116	0.475	0.736
miR-1228-3p + miR-133a-3p + miR-545-3p + miR-181a-5p	0.614	0.089	0.491	0.737
miR-1228-3p + miR-133a-3p + miR-545-3p + miR-361-5p	0.640∗	0.037	0.516	0.764
miR-1228-3p + miR-133a-3p + miR-181a-5p + miR-361-5p	0.663∗	0.015	0.538	0.789
miR-1228-3p + miR-545-3p + miR-181a-5p + miR-361-5p	0.607	0.112	0.477	0.737
miR-133a-3p + miR-545-3p + miR-181a-5p + miR-361-5p	0.623	0.067	0.493	0.753
miR-1228-3p + miR-133a-3p + miR-545-3p + miR-181a-5p + miR-361-5p	0.623	0.067	0.493	0.753

Note: ∗*P* <0.05, ∗∗*P* <0.01.

## Data Availability

The data used to support the findings of this study are available from the corresponding author upon request.

## Abbreviations

NSCLC: Non-small cell lung cancer
miRNAs: microRNAs
GEO: Gene expression omnibus
DEmiRNAs: Differentially expressed miRNAs
RRA: Robust rank Aggreg
RT-qPCR: Reverse transcription- quantitative polymerase chain reaction
ROC: Receiver operating characteristic
AUC: Area under the curve
CI: Confidence interval
LDCT: Low-dose computed tomography
KEGG: Kyoto encyclopedia of genes and genomes
OS: Overall survival
SD: Standard deviation
ADC: Adenocarcinoma
SCC: Squamous cell carcinoma
HR: Hazard ratio.
